# Efficient Distributed Wireless Power Transfer System for Multiple Wearable Sensors through Textile Coil Array

**DOI:** 10.3390/s23052810

**Published:** 2023-03-03

**Authors:** Zuolin Li, Junhyuck Lee, Jaemyung Lim, Byunghun Lee

**Affiliations:** 1 School of Electronic Engineering, Hanyang University, Seoul 04763, Republic of Korea; 2 School of Biomedical Engineering, Hanyang University, Seoul 04763, Republic of Korea

**Keywords:** WPT, parallel resonant circuit, wearable device, textile coil

## Abstract

When it is necessary to detect various physiological signals of the human body, clothing embroidered with near-field effect patterns can be used as a long-term power supply medium to supply power to long-distance transmitters and receivers to form a wireless power supply system. The proposed system uses an optimized parallel circuit to achieve a power transfer efficiency of more than five times higher than that of the existing series circuit. The power transfer efficiency of simultaneously supplying energy to multiple sensors is increased higher than five times and even more when only one sensor is coupled. When powering eight sensors at the same time, the power transmission efficiency can reach 25.1%. Even when eight sensors powered by the coupled textile coils are reduced to one, the power transfer efficiency of the whole system can reach 13.21%. Additionally, the proposed system is also applicable when the number of sensors ranges from 2 to 12.

## 1. Introduction

Wearable devices [1,2,3,4], which collect various physiological information about the human body are widely used in many areas, such as medical devices, sports, and human–machine interface research [5]. In those areas, there are huge demands on placing multiple sensors (as receivers) on various parts of the human body to simultaneously collect human activity. Since all the distributed sensors [6,7,8,9,10] have to be powered to operate, a separate power source, including batteries, supercapacitors, fuel cells, solar cells, and generators, can be used with the sensor [11]. To avoid bothering the normal activities of the human body the power supply device is required to have high flexibility and strong mechanical strength. However, battery-powered wearable devices, the most widely used type of distributed sensors, need to have their battery replaced or recharged after a certain period, and the battery increases the weight and volume [12]. The problem of requiring a periodic replacement can be solved by adopting the energy harvester to construct a self-recharging power system for a continuous power supply [13,14]. However, using clothes embroidered with inductive circuits made of wires as a medium to wirelessly power multiple sensors [15,16] can also effectively solve the problem of high battery cost and need for regular replacement, and does not require complex technologies.

**Figure 1:**
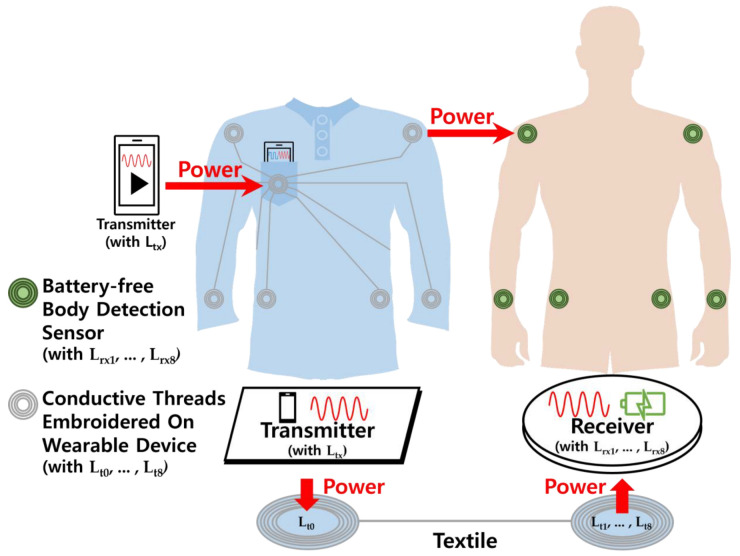
A wireless power supply system consisting of TX, the textile embroidered with induction patterns made of wires, and RX (sensor).

In [17], a system using clothes embroidered with sensing circuits made of wires to power multiple sensors was proposed. However, it wastes power on the coil, which is not coupled, and reduces the power transfer efficiency (PTE) because the coils are connected in series. As shown in Figure 1, we propose a system that uses an optimized parallel circuit to improve the PTE of the whole system. Even if the number of sensors coupled by the textile is reduced in the proposed system, the PTE of the entire circuit will not be as low as that of [17]. In addition, the proposed system utilizes the capacitors of the transmitter (TX) and receiver (RX) to resonate the entire circuit, which solves the problem of low PTE.

## 2. Materials and Methods

The PTE is inversely proportional to the distance between the Tx and Rx coils, and it limits the distance between two coils to less than a few centimeters. In addition, because the textile coil is embroidered on the clothes for the convenience of cleaning and storage, and considering the comfort of wearing, capacitors cannot be added to the textile. Therefore, the PTE in the system is significantly reduced. To solve this problem, we proposed a topology that used capacitors in the TX and RX to resonate the entire circuit and improve the overall PTE. In the proposed circuit, the PTE was improved by changing the value of the capacitor *C_tx_* on TX and *C_rx_* in RX to make the whole circuit resonate without adding any components to the original TX and RX.

### 2.1. Series Circuit and Parallel Circuit

**Figure 2:**
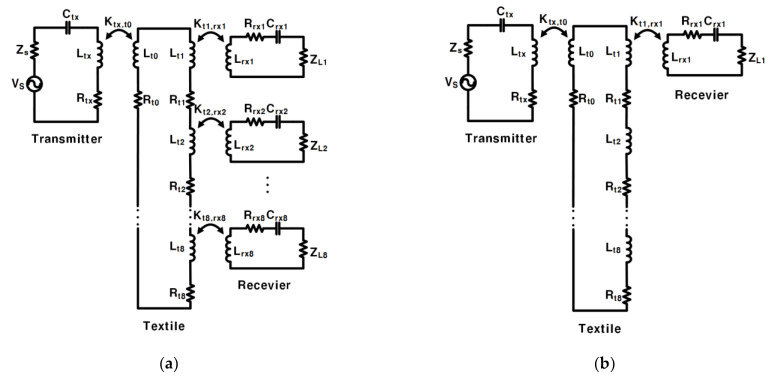

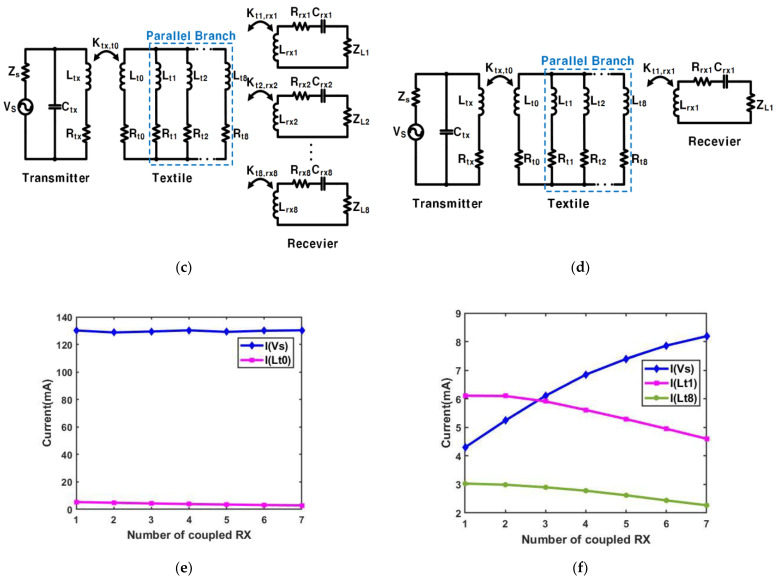
(**a**) Series circuit for the textile with 8 RXs coupled; (**b**) Series circuit for the textile with 1 RX coupled; (**c**) Parallel circuit for the textile with 8 RXs coupled; (**d**) Parallel circuit for the textile with 1 RX coupled; (**e**) The current of *V_s_* and the current of the textile coil *L_t_*_0_ in the series current when the number of coupled RXs is *n* (*n* is 1 to 8); (**f**) The current of *V_s_*, the current of the textile coil *L_t_*_1_, and the current of the textile coil *L_t_*_8_ in the parallel circuit when the number of coupled RXs is *n* (*n* ranges from 1 to 8).

The circuit in [17] consisted of a series of inductors (Figure 2a), and the circuit had a disadvantage: when the number of coupled RX was reduced without changing the textile, a significant portion of the power was wasted on textile coils that had no RX coupled. Additionally, when the series circuit did not change the textile and only one RX remained coupled (Figure 2b): the PTE was very low.

Our proposed system uses an optimized parallel circuit (Figure 2c), which solves the problem of the series circuit above. The parallel branch (the branch in the parallel circuit where the coil in the textile couples with RX), when coupled to RX, has a lower impedance than the parallel branch not coupled to RX so that most of the current flows into the parallel coupled branch. This solves the problem of wasting a lot of power on textile coils that are not coupled to RX. Additionally, without changing the textile, even if only one RX is coupled (Figure 2d), the PTE of the whole system is not low.

Figure 2e is the current of the input voltage source (*V_s_*) and the current of the textile *L_t_*_0_ in the series circuit. In this case, *n* (the number of coupled RX) RXs are coupled without changing the textile because the textile coils are connected in series, and the current of *L_t_*_1_, *L_t_*_2_, …, and *L_t_*_8_ are equal. Therefore, the current on each textile coil is equal regardless of whether the textile coil is coupled to RX or not. 

The currents on the textiles in the proposed parallel connection circuits are shown in Figure 2f. It shows the case, where *L_rx_*_1_ ~ *L_rxn_* is coupled, but *L_rxn+_*_1_ ~ *L_rx_*_8_ is not coupled. The textiles are connected in parallel, and we assumed that all the load resistance(*Z_Ln_*) was the same. Accordingly, I(*L_t_*_1_) represents the coil coupled with RX, and I(*L_t_*_8_) represents the other case. As shown in Figure 2f, the current through the textile coil *L_t_*_8_ is only half of the current through the textile coil *L_t_*_1_. Consequently, the parallel branch coupled to RX flows about the larger current twice as much compared to the parallel branch, which is not coupled to RX. Compared to the series circuit where the textile flows with the same amount of current regardless of the existence of the coupled RX, the proposed parallel circuit concentrates the current on the coupled RXs, which requires a current.

It was assumed that the maximum number of coupled RXs in the system was 8 and that (i) the parameters of each textile coil coupled with the RX coil were equal (*L_t_ = L_t_*_1_ = … = *L_t_*_8_ and *Rt* = *R_t_*_1_ = … = *R_t_*_8_). (ii) Because of the circuit characteristics, the textile coil coupled with the TX coil and the textile coil coupled with the RX coil were different *L_t_*_0_ ≠ *L_t_* and *R_t_*_0_ ≠ *Rt*. (iii) The coupling coefficients of each RX coil and the coupled textile coil are equal to *K_t_*_,*rx*_ = *K_t_*_,*rx*1_ = … = *K_t_*_,*rx*8_. (iv) Each Rx coil is the same *L_rx_* = *L_rx_*_1_ = … = *L_rx_*_8_ and *R_rx_* = *R_rx_*_1_ = … = *R_rx_*_8_. (v) The load impedance value *Z_l_* = *Z_L_*_1_ = … = *Z_L_*_8_ and capacitance *C_rx_* = *C_rx_*_1_ = … = *C_rx_*_8_ in each RX are the same. (vi) The operating resonance frequency of the whole system is *ϖ* = 13.56 MHz.

### 2.2. Circuit Design

**Figure 3:**
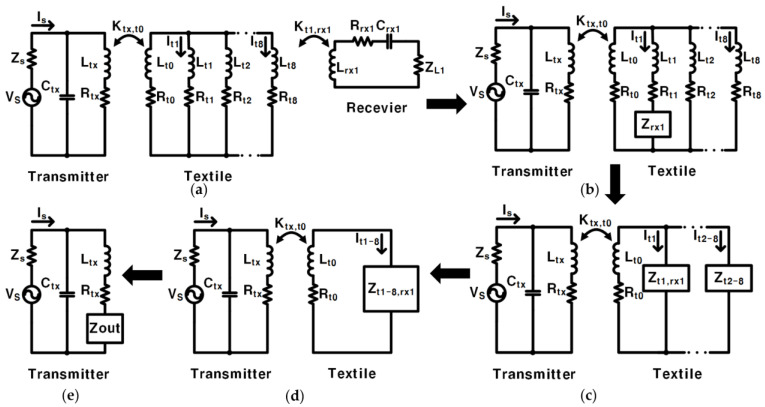
(**a**–**e**) are equivalent circuits.

The equivalent circuits of Figure 2d are shown in Figure 3 [18]. Eight textile coils can be coupled to RX, but only one textile coil, *L_t_*_1,_ is coupled to RX, and the remaining textile coils, *L_t_*_2_, *L_t_*_3_, …, and *L_t_*_8,_ are not coupled to RX. Among them, the current flowing through the uncoupled textile coils *L_t_*_2_, *L_t_*_3_, …, and *L_t_*_8_ are equal *I_t_*_2_ = *I_t_*_3_ = … = *I_t_*_8_. In [19], the equivalent impedance on RX, *Z_rx_*_1_ in Figure 3c, is derived as:

(1) $$Z_{rx1}=\frac{\omega^{2}K_{t1,rx1}{}^{2}L_{t1}L_{rx1}}{\left(j\omega L_{rx1}+R_{rx1}\right)+\frac{1}{j\omega C_{rx1}}+Z_{L1}}.$$

The equivalent impedance on the parallel branch of *L_t_*_1_, *Z_t_*_1, *rx*1_ in Figure 3c is derived as:

(2) $$Z_{t1,rx1}=\left(j\omega L_{t1}+R_{t1}\right)+Z_{rx1}.$$

The equivalent impedance on parallel branches of *L_t_*_2_, *L_t_*_3_, …, *L_t_*_8_, *Z_t_*_2*–*8_ in Figure 3c, is derived as:

(3) $$Z_{t2–8}=\frac{1}{7}\left(j\omega L_{t}+R_{t}\right).$$

The *Z_t_*_1–8,*rx*1_ in Figure 3d that is equivalent to the impedance is derived as:

(4) $$Z_{tx1–8,rx1}=\frac{1}{\frac{1}{Z_{t1,rx1}}+\frac{1}{Z_{t2–8}}}=\frac{1}{\frac{1}{\frac{1}{7}\left(j\omega L_{t}+R_{t}\right)}+\frac{1}{\left(j\omega L_{t}+R_{t}\right)+Z_{rx1}}}$$

Finally, the total equivalent impedance of the textile in Figure 3d and *Z_out_* in Figure 3e, is derived as:

(5) $$Z_{out}=\frac{\omega^{2}K_{tx,t0}{}^{2}L_{tx}L_{t0}}{\left(j\omega L_{t0}+R_{t0}\right)+Z_{t1–8,rx1}}$$

According to the above formulas, the currents in the circuit can be deduced. The current *I_s_* of *V_s_* is:

(6) $$I_{s}=\frac{V_{s}}{Z_{s}+[\frac{1}{j{\omega C}_{tx}}||\left\{\left(j\omega L_{tx}+R_{tx}\right)+Z_{out}\right\}]}$$

After that, the current of *L_tx_* can be obtained as:

(7) $$I\left(L_{tx}\right)=\frac{\left[\frac{1}{j{\omega C}_{tx}}||\left\{\left(j\omega L_{tx}+R_{tx}\right)+Z_{out}\right\}\right]}{\left(j\omega L_{tx}+R_{tx}\right)+Z_{out}}\cdot I_{s}$$

In Figure 3a, the receiver part can obtain the following equation through Kirchhoff’s voltage law:

(8) $$\left\{\left(j\omega L_{rx1}+R_{rx1}\right)+\frac{1}{j\omega C_{rx1}}+Z_{L1}\right\}\cdot I\left(Z_{rx1}\right)+j{\omega K}_{t1,rx1}\sqrt{L_{t1}L_{rx1}}\cdot I_{t1}=0$$

The relationship between *I*(*Z_rx_*_1_) and *I_t_*_1_ can be obtained as:

(9) $$I\left(Z_{rx1}\right)=-\frac{j{\omega K}_{t1,rx1}\sqrt{L_{t1}L_{rx1}}}{\left(j\omega L_{rx1}+R_{rx1}\right)+\frac{1}{j\omega C_{rx1}}+Z_{L1}}\cdot I_{t1}$$

In Figure 3d, the textile part can obtain the following equation through Kirchhoff’s voltage law:

(10) $$\left\{\left(j\omega L_{to}+R_{t0}\right)+Z_{t1–8,rx1}\right\}\cdot I_{t1–8}+j{\omega K}_{tx,t0}\sqrt{L_{tx}L_{t0}}\cdot I\left(L_{tx}\right)=0$$

The relationship between *I_t_*_1*–*8_ and I(*L_tx_*) can be obtained as:

(11) $$I_{t1–8}=-\frac{j{\omega K}_{tx,t0}\sqrt{L_{tx}L_{t0}}}{\left(j\omega L_{to}+R_{t0}\right)+Z_{t1–8,rx1}}\cdot I\left(L_{tx}\right)$$

Because it is a parallel circuit, the relationship between *I_t_*_1_ and *I_t_*_1*–*8_ is:

(12) $$I_{t1}=\frac{Z_{t1–8,rx1}}{Z_{t1,rx1}}\cdot I_{t1–8}$$

Substituting Formulas (11) and (12) into Formula (9) results in:

(13) $$\begin{array}{l} I\left(Z_{rx1}\right)=\\ -\frac{\omega^{2}K_{tx,t0}K_{t1,\mathrm{rx}1}\sqrt{L_{tx}L_{t0}}\sqrt{L_{t1}L_{rx1}}}{\left\{\left(j\omega L_{to}+R_{t0}\right)+Z_{t1–8,rx1}\right\}\cdot \left\{\left(j\omega L_{rx1}+R_{rx1}\right)+\frac{1}{j\omega C_{rx1}}+Z_{L1}\right\}}\cdot \frac{Z_{t1–8,rx1}}{Z_{t1,rx1}}\cdot I\left(L_{tx}\right) \end{array}$$

Substituting Formulas (7) and (13) can obtain the relationship between *I*(*Z_rx_*_1_) and *Is*. The value of *I*(*Z_rx_*_1_) can be obtained by calculating the value of *Is* through the formula (6). The RMS of the input power *P_in_* is:

(14) $$P_{in}=\frac{1}{\sqrt{2}}\left|V_{s}\right|\cdot \frac{1}{\sqrt{2}}\left|I_{s}\right|$$

The RMS of the output power *P_out_* is:

(15) $$P_{out}={\left\{\frac{1}{\sqrt{2}}\left|I\left(Z_{rx1}\right)\right|\right\}}^{2}\cdot Z_{rx1}$$

Therefore, the PTE *η* is:

(16) $$\eta =\frac{P_{out}}{P_{in}}\cdot 100\%$$

Using this equivalent circuit model can make subsequent calculations and understanding more convenient.

#### 2.2.1. Resonance of Parallel Branche

To couple the current of the textile coil to an RX (*I_t_*_1_) much larger than the current of the textile coil not coupled to RX (*I_t_*_8_), the imaginary part of the parallel branch impedance where *L_t_*_1_ is located has to be 0 [20]. Therefore, the following in Equation (17) has to be met.

(17) $$Im\left(Z_{t1,rx1}\right)=Im\{\left(j\omega L_{t1}+R_{t1}\right)+Z_{rx1}\}=0$$

The value of *C_rx_*_1_ can be calculated by Equation (17).

**Figure 4:**
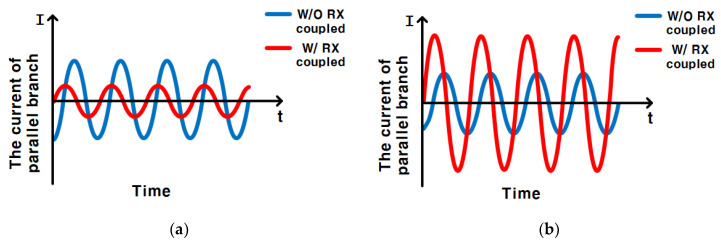
(**a**) When *C_rx_*_1_ and *L_rx_*_1_ resonate in the RX, they are the current of the parallel branch not coupled to RX, and the current of the parallel branch coupled to RX; (**b**) When *C_rx_*_1_ resonates to the parallel branch of *L_t_*_1_ coupled to RX, they are the current of the parallel branch not coupled to RX and the current of the parallel branch coupled to RX.

Figure 4a shows the current of the parallel branch which is not coupled to RX (the parallel branch of *L_t_*_2_, *L_t_*_3_, *L_t_*_4_, *L_t_*_5_, *L_t_*_6_, *L_t_*_7_, *L_t_*_8_), and the current of the parallel branch coupled to RX (the parallel branch of *L_t_*_1_) when *C_rx_*_1_ and *L_rx_*_1_ resonate in the RX. The current of the parallel branch not coupled to RX was greater than the current of the parallel branch coupled to RX, which causes most of the power to be consumed in the textile coils not coupled to RX.

Figure 4b is the current of the parallel branch not coupled to RX and the current of the parallel branch coupled to RX when *C_rx_*_1_ makes the parallel branch of *L_t_*_1_ resonate. More current flows into the coupled textile coil due to the resonance of the coupled parallel branch, and it reduces unnecessary current consumption. The current of the parallel branch not coupled to RX is smaller than the current of the parallel branch coupled to RX, which will reduce the power wasted on the textile coils not coupled to the RX coil, and this improves the PTE of the entire system.

#### 2.2.2. Resonance of the Whole Circuit

To improve the PTE of the whole system when one RX is coupled, the capacitor *C_tx_* in the TX resonates with the entire circuit [21]. When an RX is coupled, the imaginary part of the entire circuit impedance is 0.

(18) $$Im\left[\frac{1}{j\omega C_{tx}}∥\left\{\left(j\omega L_{tx}+R_{tx}\right)+Z_{\mathrm{out}}\right\}\right]=0$$

Then, the value of *C_tx_* is derived as:

(19) $$C_{\mathrm{tx}}=\frac{1}{\omega \cdot Im\{\left(j\omega L_{tx}+R_{tx}\right)+Z_{out}\}}.$$

#### 2.2.3. Textile Coil Value and PTE

The ratio of the current *I_t_*_1_ flowing through *L_t_*_1_ to the current *I_t_*_8_ flowing through *L_t_*_8_ in Figure 3b is:

(20) $$\frac{I_{t1}}{I_{t8}}=\frac{j\omega L_{t}+R_{t}}{\left(j\omega L_{t}+R_{t}\right)+Z_{rx1}}$$

**Figure 5:**
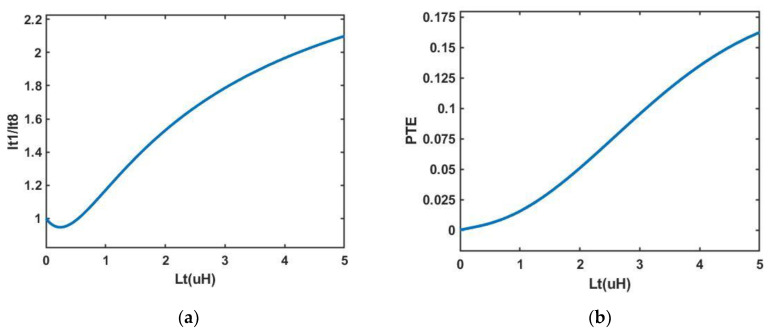
(**a**) The relationship between *L_t_* (the inductance of textile coil) and the current ratio *I_t_*_1_/*I_t_*_8_; (**b**) The relationship between *L_t_* and PTE.

Figure 5a demonstrates the linear relationship between *I_t_*_1_/*I_t_*_8_ in regards to *L_t_*. When the value of *L_t_* is larger, the value of *I_t_*_1_/*I_t_*_8_ is larger. Then, more current flows into the textile coil *L_t_*_1_ coupled to RX, and this reduces unnecessary power loss. The ratio of the current *I_t_*_1_ flowing through *Z_t_*_1, *rx*1,_ and the current *I_t_*_2–8_ flowing through *Z_t_*_2–8_ in Figure 3c is derived as:

(21) $$\frac{I_{t1}}{I_{t2–8}}=\frac{Z_{t2–8}}{Z_{t1,rx1}}=\frac{\frac{1}{7}\left(j\omega L_{t}+R_{t}\right)}{\left(j\omega L_{t}+R_{t}\right)+Z_{rx1}}$$

Equation (21) shows that the value of *I_t_*_1_/*I_t_*_2–8_ is larger when the value of *L_t_* is larger. It means that more current flows into the textile coil in which *L_t_*_1_ is coupled with RX when *L_t_* increases.

When the value of *L_t_* increases, the value of *Z_t_*_1–8,*rx*1_ should increase as well. It can be seen from formula (4) that *Z_t_*_1–8,*rx*1_ is smaller than 1/8*L_t_*, and 1/8*L_t_* is smaller than *L_t_*_0_. Therefore, when the value of the current *I_t_*_1–8_ is reduced, the power consumed in the textile coil *L_t_*_0_ is reduced, thereby increasing the PTE of the whole system [22,23,24,25]. Figure 5b shows the linear relationship between *L_t_* and the PTE of the whole system, which can confirm the previous inference that the PTE of the whole system increases with the increment of *L_t_*.

After many calculations and simulations, we found that, unlike the series circuit, when the impedance of the textile coil *L_t_*_0_ coupled with the TX coil and the textile coil *L_t_*_1_, …, *L_t_*_8_ coupled with the RX coil in the parallel circuit is the same, the PTE is reduced. Although the value of *Z_t_*_1–8,*rx*1_ increases as the value of *Lt* increases, the value of *Z_t_*_1–8,*rx*1_ is small because of the parallel structure. If the value of *Z_t_*_1–8,*rx*1_ of the part coupled with RX is small, the power consumed by the textile coil coupled with TX increases, resulting in a large amount of power being wasted in the textile coil coupled with TX. Therefore, *L_t_*_0_ is much smaller than *L_t_* in the textile part of the proposed system.

### 2.3. Proposed Circuit with PA

Class-D power amplifiers (Class-D PA) [26,27] are switching amplifiers that can provide 100% efficiency under ideal conditions, but the parasitic capacitance and on-resistance in the crystal switch reduce the actual efficiency. The current mode Class-D amplifier (CMCD) in [28] proposed zero-voltage switching (ZVS) to improve efficiency. This type of amplifier can take advantage of the drain capacitance of the circuit that loads the output so that it can be used in high-frequency circuits.

**Figure 6:**
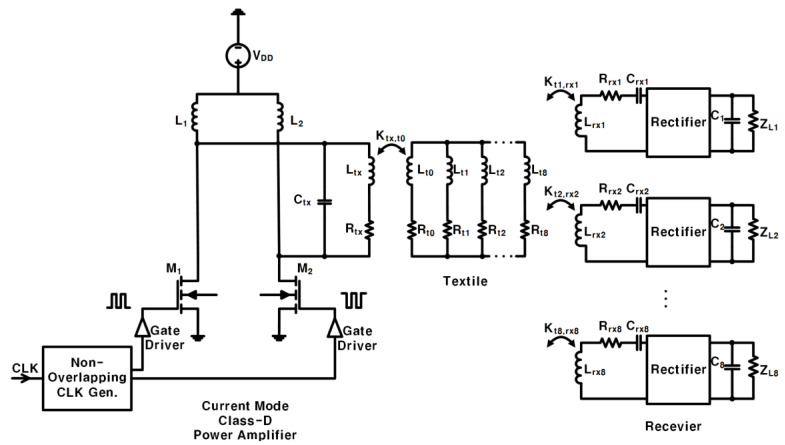
The designed circuit uses the CMCD.

The CMCD uses a choke inductance to convert the DC voltage source *V_DD_* into an equivalent DC current source [29,30]. A non-overlapping clock generator and gate driver were used to provide complementary signals for two switch mode transistors (M1 and M2). Two complementary switch-mode transistors and a filter transformed the input current into a sine wave. By using the CMCD shown in Figure 6, even if the value of the input power supply voltage *V_DD_* is small, relatively high power is delivered to the load (PDL) and can be obtained. Therefore, the problem of high PTE and low PDL of the above-mentioned system is solved.

## 3. Results

**Figure 7:**
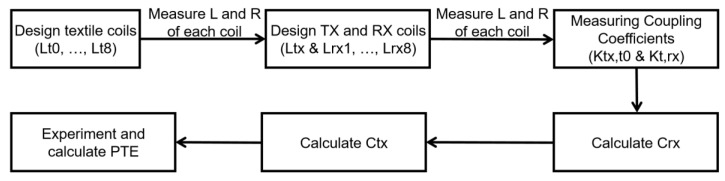
Design and experimental process.

The design and experimental process are shown in Figure 7. The inductance L, parasitic resistance R, and the coupling coefficients of the designed textile TX & RX coils are measured and summarized in Table 1. Based on the measured values, the required capacitance of *C_rx_* in RX and *C_tx_* in TX for resonance was calculated. Then, we adjusted the capacitances while running the experiment, and PTE was measured based on adjusted values.

### 3.1. Characteristics of Designed Coils

**Figure 8:**
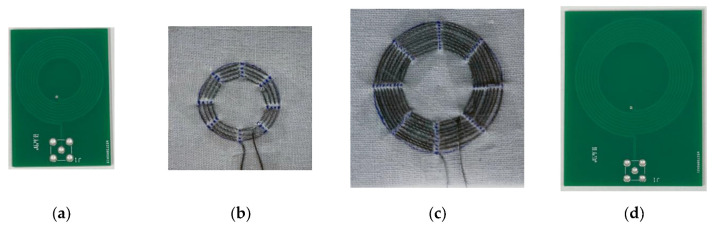
(**a**) TX Coil *L_tx_*; (**b**) Textile Coil *L_t_*_0_; (**c**) Textile Coil *L_t_*; (**d**) RX coil *L_rx_*.

The designed TX coils, *L_tx_*, are shown in Figure 8a [31]. Figure 8b shows the designed textile coil *L_t_*_0_, which was coupled with the TX coil [32,33,34], and Figure 8c shows the designed textile coil *L_t_*_1_, which was coupled with the RX coil (Figure 8d). The specifications of the proposed system are summarized in Table 1, including the values of the TX coil, textile coil, and RX coil.

After several calculations and simulations, when the coupling coefficient *k* was less than a certain value, *C_rx_* was not able to reach the imaginary impedance of the coupled parallel branch zero. If *C_rx_* cannot reach the imaginary impedance of the coupled parallel branch zero, the optimum value of PTE is lower. In order to obtain an optimally high PTE, the value of *k* needs to be as high as possible. Therefore, when designing the TX coil, RX coil, and textile coil, in order to increase the value of *k*, the outer diameter of the TX coil has to be equal to the outer diameter of the textile coil coupled with it, and the outer diameter of the RX coil has to be equal to the outer diameter of the textile coil coupled with it.

### 3.2. Experiment

**Figure 9:**
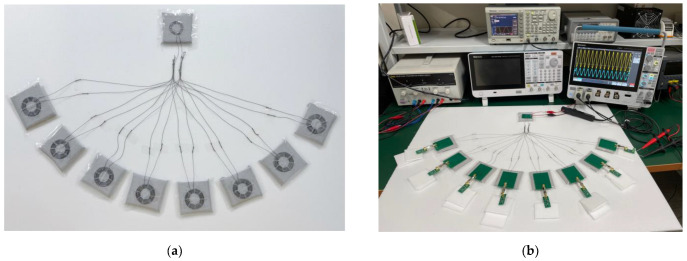

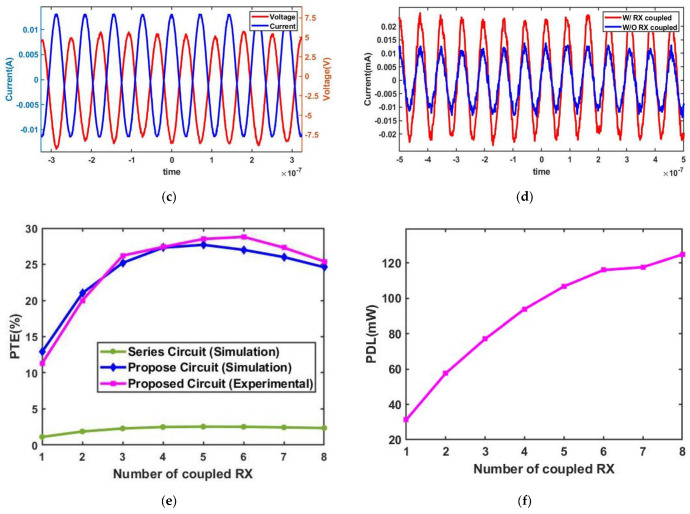
(**a**) The textile part; (**b**) The experiment with function; (**c**) The voltage and current are in-phase when only one RX is coupled; (**d**) The current of the parallel branch coupled with RX and the current of the parallel branch not coupled with RX; (**e**) The PTE of serial simulation results, proposed system simulation results and proposed system experimental results; (**f**) Using CMCD to power *n* (*n* = 1 to 8) RX, the PDL of RX.

The proposed system composed of designed textile coil connections is shown in Figure 9a. We used stainless steel wire with a diameter of 0.25 mm and a resistance of 0.83 Ω/inch. The distance between the TX coil and the textile coil coupled to the TX coil was 0.07 mm, and the distance between the RX coils and the textile coils coupled to the RX coils was also 0.07 mm. The distance between each textile coil coupled to the TX coil and the textile coil coupled to the RX coil was 30 cm. The wires connecting the TX-coupled textile coil and the RX-coupled textile coil also contributed to resistance, and the resistance of each 30 cm wire was 15 Ω.

Figure 9b shows the experimental setup that used a function generator as a power supply. Figure 9c shows the output voltage and the current of the power supply when only one RX is coupled. Since the measured output voltage and current of the power supply are in phase, it verifies that the circuit is in resonance. Figure 9d shows the measured current of the parallel branch with coupled RX and the current of the parallel branch without coupled RX. It can be seen that the current of the parallel branch with coupled RX was about twice as large compared to that of the parallel branch without coupled RX, and most of the current flow into the parallel branch was with coupled RX. The power consumed by the uncoupled textile coil was about 1/4 of the power consumption of the coupled textile coil. It indicates that most of the power was consumed by the coupled textile coil, and unnecessary loss was reduced.

Figure 9e shows the PTE of the simulation of the series circuit (prior work) and the PTE of the simulation and experiment of the proposed circuit. In both a series circuit and the proposed optimized parallel circuit, the PTE with only one RX coupling was significantly lower compared to the case where multiple RXs were coupled. When the number of coupled RX was reduced, the power dissipated in the textile coils that were not coupled with RX. When only one RX was coupled, the other seven uncoupled textile coils wasted a lot of power. The parallel optimization method we proposed made sure that most of the current flow into the textile coil was coupled with the RX regardless of how many RXs were coupled. Accordingly, the current flowing into the textile coil without coupling RX was reduced, where unnecessary power was lost. In addition, when only one RX was coupled, the whole circuit resonated, and this further improved the PTE.

The PTE of the series circuit coupled with one RX in the simulation was only 1.12%, while the PTE of the proposed parallel circuit was 12.9% in the simulation and 11.3% in the experiment. In the simulation, the PTE when the series circuit coupled 3~8 RXs was 2.3~2.55%. When the proposed optimized circuit coupled 3~8 RXs, the PTE was 24.6~27.7% in the simulation and 25.4~28.8% in the experiment. It proves that the proposed method maintains PTE independent of how many RX are coupled, and the PTE is 10 times higher than that of the series circuit. Figure 9f shows the measured PDL when the CMCD powers the proposed circuit and PDL increases.

**Figure 10:**
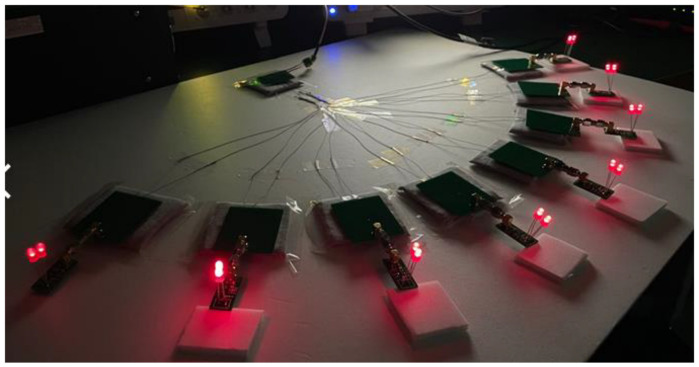
TX powers 8 RXs with LEDs through textile.

Through the textile circuit, power was supplied from TX to RX. Eight RXs with LEDs (each RX has two LEDs) were used as an alternative to the implantable sensors on human skin (Figure 10). It shows the TX can supply 8 RXs with a distance of 30cm without any issues.

## 4. Discussion

**Figure 11:**
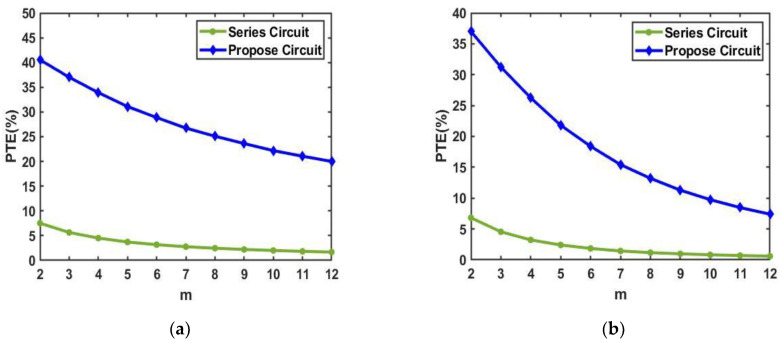
(**a**) PTE of the series circuit and the proposed circuit when *n* = *m* and (**b**) When *n* = 1.

The number of coils that can be coupled to RX in the textile is *m*, and the number of coupled RXs is *n* (*n* = 1, 2, …, *m*). We described that the number of coils m that could be coupled to the RX in the textile was eight, and the number of coupled RXs was *n* (*n* = 1, 2, …, 8). The proposed parallel optimization system could also significantly improve the PTE when *m* = 2, 3, …, 12. Figure 11a,b are the series circuit and proposed optimized parallel circuit for this case, respectively. The proposed circuit requires the whole circuit to resonate so that *C_tx_* is different for different *m*.

Figure 11a shows the simulation results of PTE when *m* = 2, 3, …, 12, and *n* = *m*, and all the coils on the textile that could be coupled to RX were coupled to RX. As m increases, the PTE of both the series connection and the proposed circuit decreases. When *m* = 2, the PTE of the series circuit was 7.52%, and the PTE of the proposed circuit was 40.54%. When *m* = 12, the PTE of the series circuit was only 1.69%, while the PTE of the proposed circuit could be maintained at 20%. The PTE of the proposed circuit was at least five times higher than that of the series circuit, and the proposed circuit could still maintain a higher PTE as the value of m increased.

Figure 11b depicts the simulation results of PTE when *m* = 2, 3, …, 12, and *n* = 1, and the textile is not changed while only one RX is coupled. When *m* = 2, the PTE of the series circuit was 6.81%, and the PTE of the proposed circuit was 37%. The PTE of the proposed circuit was more than five times that of the series circuit. With the increase in m, the PTE of the series circuit was lower than 1% and converged to zero. When *m* = 12, the PTE of the series circuit was only 0.6%. However, the proposed circuit could also maintain a PTE of 7.4% when *m* = 12. The PTE of the proposed circuit was more than 10 times that of the series circuit. Accordingly, the proposed circuit not only improved the PTE when *n* = *m* but also significantly improved the PTE when only one RX was coupled.

## 5. Conclusions

In this paper, an optimized parallel resonant system that was capable of simultaneously powering multiple sensors implanted in the human body has been proposed. The proposed structure can efficiently transmit wireless power to multiple sensors without modifying the textile coil structure while the number of coupled sensors is abruptly changed. Thanks to the automated resonant technique and the use of a parallel-based structure, the higher PTE of the entire system is maintained compared to the conventional series-based resonant system. In the system, a maximum of eight wirelessly powered sensors via textile coils were demonstrated, while the proposed technique could be extended to a large number of sensors.

## Figures and Tables

**Table 1:** Proposed system specifications.

Parameters	Value
TX coil (*L_tx_*)	Inductance = 1.66 μH Parasitic resistance = 2.07 Ω Quality factor = 68.3 Outer diameter = 28 mm Number of turns = 8
RX coil (*L_rx_*_1_, *L_rx_*_2_, *L_rx_*_3_, *L_rx_*_4_, *L_rx_*_5_, *L_rx_*_6_, *L_rx_*_7_, *L_rx_*_8_)	Inductance = 3.05 μH Parasitic resistance = 2.92 Ω Quality factor = 90 Outer diameter = 40 mm Number of turns = 8
Textile coil coupled to TX coil (*L_t_*_0_)	Inductance = 0.791 μH Parasitic resistance = 10.2 Ω Quality factor = 6.61 Outer diameter = 28 mm Number of turns = 4
Textile coil coupled to RX coil (*L_t_*_1_, *L_t_*_2_, *L_t_*_3_, *L_t_*_4_, *L_t_*_5_, *L_t_*_6_, *L_t_*_7_, *L_t_*_8_)	Inductance = 3.94 μH Parasitic resistance = 30 Ω Quality factor = 11.19 Outer diameter = 40 mm Number of turns = 9
Source impedance (*Z_s_*)	50 Ω
Load impedance (*Z_l_*)	36 Ω
Capacitance of *C_tx_*	96.3 pF
Capacitance of *C_rx_*	80.6 pF
Coupling coefficient of TX coil and textile coil (*K_tx_*_,*t*0_)	0.5
Coupling coefficient of RX coil and textile coil (*K_t_*_,*rx*_)	0.7

## Data Availability

All data is contained within the article.

## References

[1] Lin R., Kim H.J., Achavananthadith S. (2020). Wireless battery-free body sensor networks using near-field-enabled clothing. Nat. Commun..

[2] Pantelopoulos A., Bourbakis N.G. (2010). A Survey on Wearable Sensor-Based Systems for Health Monitoring and Prognosis. IEEE Trans. Syst. Man Cybern. Part C.

[3] Zhu Z., Liu T., Li G., Li T., Inoue Y. (2015). Wearable Sensor Systems for Infants. Sensors.

[4] Jung H., Lee B. (2021). Wireless Power and Bidirectional Data Transfer System for IoT and Mobile Devices. IEEE Trans. Ind. Electron..

[5] Mardonova M., Choi Y. (2018). Review of Wearable Device Technology and Its Applications to the Mining Industry. Energies.

[6] Wang C., Hwang D., Yu Z. (2013). User-interactive electronic skin for instantaneous pressure visualization. Nat. Mater..

[7] Webb R., Bonifas A., Behnaz A. (2013). Ultrathin conformal devices for precise and continuous thermal characterization of human skin. Nat. Mater..

[8] Gao W., Emaminejad S., Nyein H. (2016). Fully integrated wearable sensor arrays for multiplexed in situ perspiration analysis. Nature.

[9] Mukhopadhyay S.C. (2015). Wearable Sensors for Human Activity Monitoring: A Review. IEEE Sens. J..

[10] Majumder S., Mondal T., Deen M.J. (2017). Wearable Sensors for Remote Health Monitoring. Sensors.

[11] Gao J., Shang K. (2021). Material and configuration design strategies towards flexible and wearable power supply devices: A review. J. Mater. Chem. A.

[12] Lee Y.-H., Kim J.-S., Noh J. (2013). Wearable Textile Battery Rechargeable by Solar Energy. Nano Lett..

[13] Luo J., Wang Z.L. (2019). Recent advances in triboelectric nanogenerator based self-charging power systems. Energy Storage Mater..

[14] Xue X., Wang S., Guo W., Zhang Y., Wang Z.L. (2012). Hybridizing Energy Conversion and Storage in a Mechanical-to-Electrochemical Process for Self-Charging Power Cell. Nano Lett..

[15] Lee J., Bae B., Kim B., Lee B. (2022). Full-duplex enabled wireless power transfer system via textile for miniaturized IMD. Biomed. Eng. Lett..

[16] Komolafe A. (2021). E-Textile Technology Review–From Materials to Application. IEEE Access.

[17] Kim H.-J., Lin R., Achavananthadith S., Ho J.S. Near-field-enabled Clothing for Wearable Wireless Power Transfer. Proceedings of the 2020 IEEE Wireless Power Transfer Conference (WPTC).

[18] Harrison R.R. Designing Efficient Inductive Power Links for Implantable Devices. Proceedings of the 2007 IEEE International Symposium on Circuits and Systems.

[19] Schormans M., Valente V., Demosthenous A. (2018). Practical Inductive Link Design for Biomedical Wireless Power Transfer: A Tutorial. IEEE Trans. Biomed. Circuits Syst..

[20] Kim J., Son H.-C., Kim K.-H., Park Y.-J. (2011). Efficiency Analysis of Magnetic Resonance Wireless Power Transfer With Intermediate Resonant Coil. IEEE Antennas Wirel. Propag. Lett..

[21] Kwon H., Lee K.-H., Lee B. (2020). Inductive Power Transmission for Wearable Textile Heater using Series-None Topology. Electronics.

[22] Zhong W.X., Hui S.Y.R. (2015). Maximum Energy Efficiency Tracking for Wireless Power Transfer Systems. IEEE Trans. Power Electron..

[23] Aditya K., Williamson S.S. (2017). A Review of Optimal Conditions for Achieving Maximum Power Output and Maximum Efficiency for a Series–Series Resonant Inductive Link. IEEE Trans. Transp. Electrif..

[24] Zargham M., Gulak P.G. (2012). Maximum Achievable Efficiency in Near-Field Coupled Power-Transfer Systems. IEEE Trans. Biomed. Circuits Syst..

[25] Low Z.N., Chinga R.A., Tseng R., Lin J. (2009). Design and Test of a High-Power High-Efficiency Loosely Coupled Planar Wireless Power Transfer System. IEEE Trans. Ind. Electron..

[26] Khan H.R., Qureshi A.R., Zafar F. (2016). Design of a broadband current mode class-D power amplifier with harmonic suppression. Analog. Integr. Circuits Signal Process..

[27] Kobayashi H., Hinrichs J.M., Asbeck P.M. (2001). Current-mode class-D power amplifiers for high-efficiency RF applications. IEEE Trans. Microw. Theory Tech..

[28] Gupta A., Arondekar Y., Ravindranath S.V.G., Krishnaswamy H., Jagatap B.N. A 13.56 MHz high power and high efficiency RF source. Proceedings of the 2013 IEEE MTT-S International Microwave Symposium Digest (MTT).

[29] Long A., Yao J., Long S.I. A 13 W current mode class D high efficiency 1 GHz power amplifier. Proceedings of the 45th Midwest Symposium on Circuits and Systems, (MWSCAS-2002).

[30] El-Hamamsy S.-A. (1994). Design of high-efficiency RF Class-D power amplifier. IEEE Trans. Power Electron..

[31] Raju S., Wu R., Chan M., Yue C.P. (2014). Modeling of Mutual Coupling Between Planar Inductors in Wireless Power Applications. IEEE Trans. Power Electron..

[32] Fadhel Y.B., Bouattour G., Bouchaala D., Rahmani S., Kanoun O., Derbel N. An optimized wearable coil for Wireless Power Transfer Applications. Proceedings of the 2020 17th International Multi-Conference on Systems, Signals & Devices (SSD).

[33] Sun D., Chen M., Podilchak S. (2020). Investigating flexible textile-based coils for wireless charging wearable electronics. J. Ind. Text..

[34] Wagih M., Komolafe A., Zaghari B. (2020). Dual-Receiver Wearable 6.78 MHz Resonant Inductive Wireless Power Transfer Glove Using Embroidered Textile Coils. IEEE Access.

